# Companionship utilization and its associated factors during childbirth among mothers who delivered at public hospitals in Shashemene town, Southern Ethiopia: A mixed-method study

**DOI:** 10.1371/journal.pone.0347161

**Published:** 2026-04-28

**Authors:** Dedefo Gemeda Anbesa, Abdene Weya Kaso, Gebi Agero, Girma Bacha, Ayanos Taye

**Affiliations:** 1 Department of Midwifery, College of Health Science, Madda Walabu University, Shashemene, Ethiopia; 2 Department of Public Health, College of Health Science, Arsi University, Asella, Ethiopia; 3 Department of Health System and Policy, Institute of Health, Jimma University, Jimma, Ethiopia; 4 School of Nursing, Institute of Health, Jimma University, Jimma, Ethiopia; University of Nigeria - Enugu Campus, NIGERIA

## Abstract

**Background:**

Childbirth companionship has multiple benefits and is associated with improved maternal and neonatal outcomes. However, childbirth companionship is an important component of respectful maternity care that is still underutilized in the clinical setting. Therefore, this study assessed factors associated with childbirth companionship utilization among mothers who delivered at a public hospital in Shashemene town, southern Ethiopia.

**Methods:**

A facility-based mixed methods study was performed among 411 mothers who delivered at Public Hospitals in Shashemene town from August 20 to September 20, 2021. We entered the collected data into Epidata version 3.1 and exported it to SPSS software version 23 for analysis. A binary logistic regression model was employed to assess the relationship between independent variables and childbirth companionship utilization. A p < 0.05, with an Adjusted Odds Ratio (AOR) and 95% confidence interval (CI) were employed to declare the association. For an in-depth interview, the interview guide was utilized to collect data. We used Open Code software 4.03 for verbatim transcription, translation, and thematic analysis of the data.

**Results:**

The magnitude of childbirth companionship utilization was 14.4% (95% CI: 11.2–17.6). Having complications during pregnancy and labor [AOR = 3.25, (95% CI: 1.74, 6.05)], having a future desire to have companions [AOR = 4.02, (95% CI: 1.72: 1.66, 9.41)], primiparous [AOR = 2.54, (95% CI: 1.25, 5.28)], and good knowledge of companionship [AOR = 3.00, (95% CI: 1.53, 5.91)] were significantly associated with childbirth companionship utilization. Qualitative findings also revealed that healthcare providers’ denial, overcrowding of the delivery room, and mothers’ fear of exposing partner/family members to stress were barriers to utilizing childbirth companionship.

**Conclusion:**

In this study, the magnitude of childbirth companionship utilization was low and below the WHO recommendation. Therefore, the study highlights the significance of acting on the health system, client, and provider-side factors, which are important to improve the implementation of respectful maternity care components at the facility level. In addition, the integration of respectful maternity care counseling into routine prenatal care, regardless of mothers’ pregnancy risk status, could enhance utilization. Moreover, addressing barriers to companionship utilization through healthcare provider training, developing clear facility-based guidelines, and improving health facilities’ infrastructure is crucial to enhance childbirth companionship and improve maternal health outcomes.

## Background

Childbirth in health facilities is a critical life event that needs quality maternal healthcare services [1]. Despite advances in maternal and child healthcare services reduced maternal and neonatal death worldwide [2], the quality of maternity care remains a problem in low- and middle-income countries (LMICs) [3]. One of the important components of respectful and high-quality maternity care that is still underutilized is companionship during childbirth [4]. Birth companionship refers to non-pharmacological components of care where a woman’s selected individual, such as a partner, friend, or family member, continuously provides emotional, informational, and physical support to a woman throughout labor and delivery [5,6].

Evidence indicated that companionship during childbirth has multiple benefits and is associated with improved maternal and neonatal outcomes [7,8]. The companionship improves the relationships between couples, the bonding among family members, and increases the client satisfaction with health care providers’ services [8–10]. Besides this, the assistance offered by companions during childbirth has health benefits, such as shortening durations of labor, reducing postpartum post-traumatic stress disorder, improving quality of care, encouraging initiation of skin-to-skin contact, and breastfeeding [8,11,12]. Considering the companionship’s multiple health benefits, the World Health Organization (WHO) recommends utilizing a childbirth companionship as a main component of compassionate and respectful maternity care [13,14].

Despite the WHO recommendations, the implementation of companionship during childbirth remains limited in many settings of resource-constrained countries [15]. Evidence revealed that structural limitations within health institutions, including overcrowding, poor infrastructure, and restrictive institutional policies, often hinder the adoption of companion-friendly services [16,17]. Furthermore, social and cultural norms and misconceptions about the role of companions among mothers and health professionals limit the acceptance of companionship during childbirth [18,19].

The prevalence of companionship utilization ranges from 9.2 to 37.7% in LMICs [20,21]. In addition, the magnitude of mothers utilizing childbirth companionship was 1.2 to 49% in Sub Saharan African Countries (SSA) [ 20,22–25]. In Ethiopia context, the magnitude of women supported by individuals from their social network was 13.8% in Arba Minch, and 14.6% in Debre Markos, Ethiopia [26,27]. Previous findings revealed that the practice of childbirth companionship was influenced by sociodemographic factors (i.e., age of the mother, the mothers income, low educational status) [21,24,28,29], maternal-related factors (i.e., parity, vaginal delivery, previous cesarean delivery, history of home delivery, antenatal care visits, and presence of complications during pregnancy and labor) [21,26,28,30–32]. In addition, women’s awareness [33,34] and attitude towards the benefits and role of a companion during labor [35,36] were also found to influence the companionship utilization.

Although the global and national frameworks [37–40] advocating for respectful maternity care, including childbirth companionship, evidence of the healthcare providers’ practice in the clinical area is limited, especially during the COVID-19 era. Despite the health facilities in Shashemene town playing a crucial role in providing maternity care, there is a scarcity of information on the level of implementation of childbirth companionship, which limits healthcare leaders from designing appropriate context-specific strategies that promote client-centered and respectful care. Therefore, this study determined factors associated with the childbirth companionship utilization among women who delivered at public hospitals in Shashemene town, Southern Ethiopia.

## Methods and material

### Study setting

We performed a study in public hospitals in Shashemene Town, Southern Ethiopia. Shashemene town is located 252 kilometers from Addis Ababa, Ethiopia. Shashemene town Health Office 2024 data revealed that the town has around 519,749 people, of which 256,756 are male, and 262,993 are female. Shashemene town has three Health centers and two public Hospitals. Melka Oda General Hospital and Shashemene Referral Hospital are the public hospitals found in the town. The hospital has 240 beds to deliver its healthcare services, with an average patient flow of 282 patients per day. The hospital employed 20 physicians, two gynecologists, two surgeons, eighteen midwives, and 120 nurses to provide outpatient, inpatient, maternity care, emergency services, and other services. Melka Oda Hospital has around 123 beds to offer basic healthcare services. The hospital also recruited 91 nurses, 13 midwives, 14 medical doctors, 1 gynecologist, and other medical professionals to offer the catchment population inpatient, outpatient, maternity care, emergency treatment, and other services. On average, there are 840 mothers delivered in the two facilities.

### Study design and period

We performed a facility-based cross-sectional study supplemented by a qualitative study from August 20 to September 20, 2021.

### Population

All women who delivered at Public Hospitals in Shashemene town were the source population, whereas all mothers who delivered at public hospitals in Shashemene town during the data collection period were the study population for a quantitative study.

The qualitative study included maternity care providers, women, and birth companions for in-depth interviews.

### Inclusion and Exclusion criteria

The study included mothers who delivered vaginally, whereas those who were critically ill and admitted to the facility after the second stage of labor were excluded.

### Sample size determination

The following assumptions were taken into account when computing the sample size using the single population proportion formula: 95% confidence level, 5% margin of error, 14.6% companionship utilization [27], and 10% non-response rate. In addition, variables such as desire for companionship, complications during pregnancy and labor, and parity were used to compute the sample size for the second objective. For each variable considered, sample sizes of 246, 128, and 374 were determined. The maximum sample sizes were 411 when a 10% non-response rate was added. After comparing the samples for the two objectives, the final sample size for the study was 411.

For the qualitative study, ten study participants (three mothers, three partners, and four obstetric care providers) from two public hospitals in Shashemene town were involved in interviews. The number of participants for the in-depth interview was decided based on the saturation of the required data.

### Sampling technique

The study included two public hospitals (i.e., Melka Oda Hospital and Shashemene Referral Hospital) providing delivery services purposively. Then, using the monthly delivery case load, the sample size was allocated proportionally to the respective hospital. A systematic random sampling technique was employed to select the study respondents. The first woman was selected using the lottery method on the first day of data collection.

For the qualitative study, a criteria-based purposive sampling method was used to select respondents for in-depth interviews. Mothers were selected based on their parity from those who had not participated in a quantitative study. For the in-depth interview, the childbirth companions and maternity care providers were selected based on their history of previous hospital visits and work experience, respectively.

### Study Variables

#### Dependent Variables.

Companionship utilization during childbirth (yes/no)

#### Independent Variables.

**Sociodemographic factors**: age, religion, marital status, residence, educational status, occupation, and family monthly income

**Maternal-related factors:** complication during labor and delivery, current antenatal care utilization, parity, place of last delivery, having a companion in previous delivery, and route of last delivery; knowledge, attitude, and future desire for a companion during childbirth

**Healthcare-related factors**: Sex of provider, disclosing the information, and permission.

### Data collection tool and procedure

An interviewer-administered semi-structured questionnaire was developed based on previous studies [15,16]. The questionnaire was first developed in English and translated into the local language (i.e., Amharic/Afan Oromo) and retranslated into English by a language expert for consistency. The questionnaire consists of socio-economic factors, maternal-related factors, maternity care provider-related factors, and self-reported companionship utilization during childbirth. A pretest was conducted on 5% (21) of the sample at Arsi Negelle Primary Hospital to assess the data collection tool’s simplicity, logical flow, and consistency. The data collectors were two BSc midwives who were experienced in data collection. Data was collected by interviewing women who gave birth in selected public hospitals during their exit from the postnatal ward. The supervisor coordinated and monitored the overall data collection system, and the principal investigator checked data for completeness and accuracy daily.

Data for qualitative studies were collected using an in-depth interview guide by the researcher. During the in-depth interview, the understanding of the perspective, value, and view of maternity care providers, birth companions, and women who gave birth was explored and described. The interview was conducted in Afan Oromo, lasted for 40–60 minutes, audio recorded, and supplemented by notes. The interview took place in a convenient and participant-chosen location to ensure comfort and openness. Data triangulation was ensured by interviewing maternity care providers, birth companions, and mothers who gave birth at the hospitals.

### Data analysis

Data were coded and entered into Epidata version 3.1 and exported to SPSS software version 23 for analysis. We computed the descriptive statistics and presented them using tables and figures. Binary logistic regression analysis was used to determine the relationship between independent variables and childbirth companionship utilization. Variables with a P-value < 0.25 in the bivariable logistic regression model analysis were transferred to the multivariable logistic regression analysis. A p-value of < 0.05, with an AOR and 95% CI, was employed to declare the statistical significance. Finally, we checked the fitness of the model by the Hosmer and Lemeshow goodness-of-fit test (p = 0.567).

For qualitative studies, audio recordings in Afan Oromo were transcribed verbatim and translated into English by the principal investigator. Data were analyzed using inductive thematic analysis. Codes were generated directly from respondents’ narratives through iterative reading of transcripts by the Principal Investigator. A codebook was developed and refined during analysis to ensure consistency and analytic rigor. Open Code software version 4.03 was used for analysis.

### Ethics approval and consent to participate

The study was performed in accordance with the Declaration of Helsinki. We received anethical approval from the Jimma University Institute of Health, Faculty of Health, School of Nursing Institutional Review Board (Ref no: IHRPG1/372/21). The support letter was sent to the Health Facilities Administration by Jimma University, Institute of Health. We obtained written consent from the study participant before initiating the study.

## Results

### Socio-demographic characteristics of respondents

The quantitative data included 403 participants for the interview, with a response rate of 98.1%. Two hundred thirteen (52.9%) of women were in the 25–34 age group, with a mean age of 26 years (SD ± 4.28 years). Two hundred forty-six (61%) of the respondents were urban residents, and 395 (98%) were married. Concerning educational status, 156 (38.7%) of them attended primary (1–8) school, 115(28.5%) attended secondary school, and 49(12.2%) of them had college and above education. Residence (p < 0.0122) and mothers’ education status (p < 0.002 were associated with childbirth companionship utilization (Table 1).

**Table 1:** Socio-economic characteristics of respondents in public hospitals at Shashemene town, south Ethiopia, 2021 (n = 403).

Variable	Total (%)	Companionship utilization		P value
		Yes (%)	No (%)	
Age				
<24	20(5.0)	4(20.0)	16(80.0)	0.171
25-34	274(68.0)	45(16.4)	229(83.6)	
≥35	109(27.0)	10(9.2)	99(90.8)	
Sex of healthcare provider				
Female	146(36.2)	20(13.7)	126(86.3)	0.530
Male	177(43.9)	29(16.4)	148(83.7)	
Both	80(18.9)	9(11.3)	71(88.7)	
Residence				
Urban	246(61.0)	44 (17.9)	202 (82.1)	0.012
Rural	157(39.9)	14 (8.9)	143 (91.1)	
Mother’s educational level				
No formal education	83(20.6)	7 (8.4)	76 (91.6)	0.002
Primary school	156(38.7)	14 (9.0)	142 (91.0)	
Secondary school	115(28.5)	25 (21.7)	90 (78.3)	
College and above	49(12.2)	12 (24.5)	37 (75.5)	
Occupation status				
Housewife	192(47.6)	25(13.0)	167(87.0)	0.889
Government employee	102(25.3)	16(15.7)	86(84.3)	
Merchant	74(18.4)	12(16.2)	62(83.8)	
Student	35(8.7)	5(14.3)	30(85.7)	

### Maternal-related characteristics

Out of 403 respondents, 304 (75.4%) were multiparous, 361 (89.6%) had received antenatal care (ANC) for the current pregnancy, and 34 (9.4%) of them were informed about the birth companion during the current ANC. One hundred twenty-one (30%) of mothers had complications during childbirth, and 251(62.3%) of them had a future desire to have a companion. Parity (p < 0.026), having complications during Pregnancy & Labor (p < 0.001), Future desire to have a companion (p < 0.001), and maternal knowledge of childbirth companionship (p < 0.001) were associated with companionship utilization (Table 2).

**Table 2:** Obstetric-related variables of mothers who delivered at public hospitals in Shashemene town, south Ethiopia, 2021 (n = 403).

Variable	Total (%)	Companionship utilization		P value
		Yes	No	
Current ANC history				
Yes	361(89.6)	52(16.8)	309(83.2)	0.983
No	42(10.4)	6(14.3)	36(85.7)	
Parity				
Primiparous	99(24.6)	21 (21.2)	78 (78.8)	0.026
Multiparous	304(75.4)	37 (12.2)	267 (87.8)	
Place of last delivery(n=304)				
Home	66(21.7)	5(7.6)	61(92.4)	0.140
Health facilities	238(78.3)	35(14.7)	207(85.3)	
Route of last delivery				
Vaginal delivery	265(87.2)	34(12.8)	235(87.2)	0.362
C/S	39(12.8)	7(17.9)	32(82.1)	
Complications during Pregnancy & Labor				
Yes	121(30.0)	30 (24.8)	91 (75.2)	0.001
No	282(70.0)	28 (9.9)	254 (90.1)	
Future desire to have a companion				
Yes	251(62.3)	49 (19.5)	202 (80.5)	0.001
No	152(37.7)	9 (5.9)	143 (94.1)	
Maternal Knowledge of Childbirth Companionship				
Good	187(46.4)	40 (21.4)	147 (78.6)	0.001
Poor	216(53.6)	18 (8.3)	198 (91.7)	
Maternal Attitude towards Companionship				
Positive	255(63.3)	37 (14.5)	218 (85.5)	0.930
Negative	148(36.7)	21 (14.2)	127 (85.8)	

### Maternal knowledge of companionship

**Fig 1:**
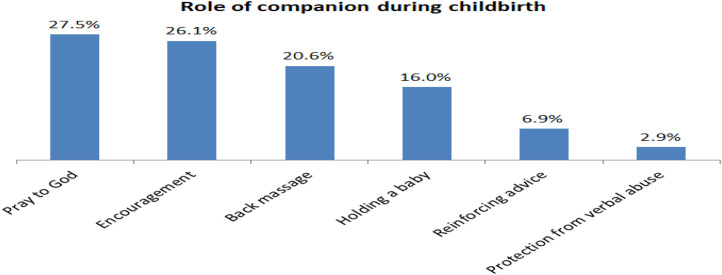
Role of companions during childbirth among mothers delivered at public hospitals in Shashemene town, 2021.

Two hundred sixteen (53.6%) of mothers had poor knowledge, and 187(46.4%) of them had good knowledge of birth companionship (Table 2). Concerning the companions’ roles during childbirth, 27.5% of mothers stated that their companion can pray for them, and 26.1% of them said they can encourage them to push. This finding is supported by qualitative findings from in-depth interviews, which found that companions have a role of providing physical support, informational support, and playing the role of advocacy during childbirth (Fig 1).

“*If companions are allowed to enter during childbirth, they may give support in different ways. They can encourage her to be strong by advising, massaging her back when she feels pain, holding her hand, and giving encouraging words to push her. In addition, for primiparous women, companions may encourage and advise her when an episiotomy is performed*” [IDI, 30 years old, Midwife]

**Fig 2:**
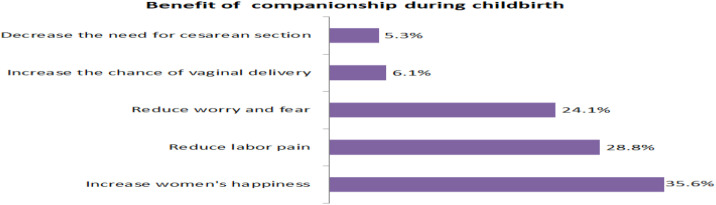
Benefit of childbirth companionship among mothers delivered at public hospitals in Shashemene town, 2021.

More than one third, 35.6% and 28.8% of mothers know that having a companion during childbirth promotes happiness and reduces labor pain, during childbirth respectively (Fig 2). This finding is supported by a qualitative study in which the majority of the key informants reported that supportive care provided by the companions reduces labor pain, avoids fear and stress, and increases the chance of vaginal delivery.

*“… Supportive care given to mothers during childbirth by their companions has a lot of benefits. They may share her pain, help her not to fear, and make her strong. Also, they may encourage mobility as it increases the chance of vaginal delivery” [IDI, 29 yrs. old midwife].*

### Maternal Attitude towards companionship during childbirth

Two hundred fifty-five (63.3%) of mothers had a positive attitude, whereas 148(36.7%) of them had a negative attitude towards companionship (Table 2). The quantitative finding is also supported by findings of interviews that having a companion during childbirth is culturally acceptable and helpful. The quantitative finding is also supported by findings of in-depth interviews that having a companion during childbirth is culturally acceptable and helpful.

*“*In *our culture, either of my family members can be with me throughout my labor and childbirth process. Nobody prohibits me from having a companion during the event, and it is socially acceptable because at that time they (the companion) want me to deliver safely and to save my life. So they don’t think about shying or embarrassing…*” [IDI, 25 yrs. old mother].

### Companionship utilization during childbirth

**Fig 3:**
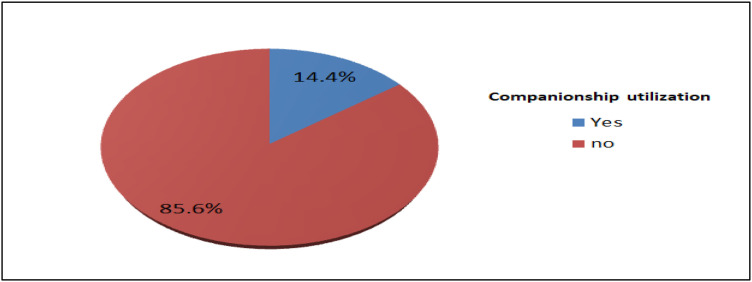
Childbirth companionship utilization among mothers who delivered at public hospitals in Shashemene town, south Ethiopia, 2021.

In this study, 58(14.4%; 95% CI: 11.2–17.6%) of mothers utilized companionship during childbirth (Fig 3).

**Fig 4:**
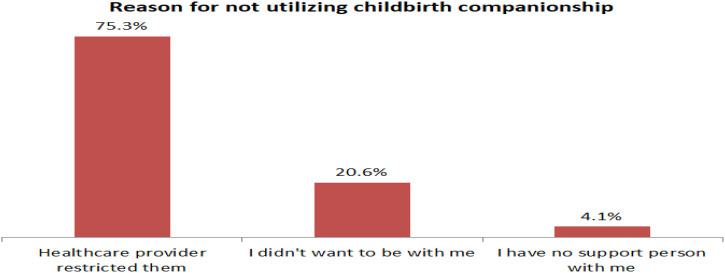
Reason for not utilizing childbirth companionship among mothers who delivered at public hospitals in Shashemene town, Ethiopia, 2021.

Three-fourths, 75.3% of mothers reported that healthcare providers’ denial was the main barrier to not utilizing childbirth companionship (Fig 4). During the in-depth interview, mothers also indicated that maternity care providers didn’t permit their companions to be with them during childbirth.

“*When I was in labo*r*, doctors didn’t allow my husband to be with me and ordered him not to enter the room at all. However, I don’t want him to be outside.*” [IDI, a 25 yrs. old Mother]

In addition, the maternity Care providers participate in in-depth interview also indicated that they don’t permit the companions to enter the labor and delivery room with the laboring mother.

“*We are happy if the family members will be with the mother and support her. But the room we have here is very narrow, and we are not allowing them to enter/be with mothers during labor and delivery due to fear of overcrowding…*” [IDI, a 30 yrs. old Midwife].

### Factors associated with childbirth companionship utilization

Variables such as age, residence, mothers’ educational level, parity, complications during pregnancy and labor, future desire to have a companion, and mothers’ knowledge of childbirth companionship were candidate variables for multivariable logistic regression. Primiparous, presence of complications during pregnancy and labor, future desire to be accompanied by a companion, and mothers’ knowledge of childbirth companionship were found to have a significant relationship with companionship utilization in multivariable logistic regression. The odds of utilizing childbirth companionship were 2.54 times higher among primiparous mothers than multiparous women [AOR = 2.54, (95% CI: 1.25, 5.28)]. In-depth interviews also revealed that obstetric care providers occasionally permit primiparous mothers to be accompanied by their companions during childbirth.

“*We allow companions to enter the delivery room with primiparous mothers. Primiparous mothers often refuse services such as performing an episiotomy or applying instrumental delivery if necessary at that time the companion may advise her” [IDI, 25-year-old midwife*].

The likelihood of utilizing childbirth companionship was 3.25 times higher among mothers who had complications during labor and pregnancy than their counterparts [AOR = 3.25, (95% CI: 1.74, 6.05)]. This finding is also strengthened by qualitative findings in which obstetric care providers stated that they permit companions to enter the delivery room to support mothers with complications during childbirth.

“…*Sometimes, for those mothers who developed complications like pre-eclampsia, PPH, APH, etc., we allow one person from her family to be with her*” [IDI, a 30-year-old midwife].

Mothers who had a future desire to utilize childbirth companionship have 4.02 times higher odds of being accompanied by their companion compared to their counterparts [AOR = 4.02, (95% CI: 1.72, 9.41)]. In addition, the odds of utilizing childbirth companionship were 3.00 times higher among women who had good knowledge of companionship than those who had poor knowledge [AOR = 3.00, (95% CI: 1.53, 5.91)](Table 3).

**Table 3:** Factors associated with childbirth companionship utilization in public hospitals, Shashemene town, Ethiopia, 2021.

Variables category	Companionship utilization		COR(95%CI)	AOR(95%CI)
	Yes (%)	No (%)		
Age				
<24	4(20.0)	16(80.0)	1	1
25-34	45(16.4)	229(83.6)	0.77 (0.25, 2.40)	0.50 (0.13, 1.89)
≥35	10(9.2)	99(90.8)	0.41 (0.11, 1.45)	0.76 (0.17, 3.42)
Residence				
Urban	44 (17.9)	202 (82.1)	2.22 (1.18,4.21)	1.10 (0.46,2.67)
Rural	14 (8.9)	143 (91.1)	1	1
Mother’s educational level				
No formal education	7 (8.4)	76 (91.6)	1	1
Primary school	14 (9.0)	142 (91.0)	1.07 (0.414,2.76)	0.50 (0.16,1.54)
Secondary school	25 (21.7)	90 (78.3)	3.02 (1.24,7.36)	1.28 (0.37,4.41)
College and above	12 (24.5)	37 (75.5)	3.52 (1.28,9.68)	1.39 (0.34,5.64)
Complications during Pregnancy & Labor				
Yes	30 (24.8)	91 (75.2)	2.99(1.69,5.28)	3.25(1.74,6.05)*
No	28 (9.9)	254 (90.1)	1	1
Future desire to have a companion				
Yes	49 (19.5)	202 (80.5)	3.85(1.84,8.09)	4.02(1.72,9.41)*
No	9 (5.9)	143 (94.1)	1	1
Parity				
Primiparous	21 (21.2)	78 (78.8)	1.94 (1.08,3.51)	2.54(1.25,5.28)*
Multiparous	37 (12.2)	267 (87.8)	1	1
Knowledge of companionship				
Good	40 (21.4)	147 (78.6)	2.99 (1.65,5.43)	3.00(1.53,5.91)*
Poor	18 (8.3)	198 (91.7)	1	1

Note:* P ≤ 0.05

## Discussion

Companionship during childbirth was recognized as an important component of respectful maternity care [41]. Therefore, this study determined factors associated with childbirth companionship utilization among women who delivered at Public hospitals in Shashemene town, Ethiopia. In this study, 14.4% of mothers were accompanied by their companions during childbirth. This finding is in line with studies done in Tanzania, 12% [23], in Arba Minch, Ethiopia, 13.8%, and Debre Markos, Ethiopia, 14.6% [26,27]. However, this finding is lower than studies done in Brazil, 32.7% [21], in Saudi Arabia, 27% [25], in South Africa, 49% [42], in Nepal, 19% [29], and in Kenya, 29% [24]. A difference in the study setting, data collection techniques, sampling methods, and eligibility requirements could account for the discrepancy. Studies from Kenya and Brazil included both private and public institutions in the study, whereas the research performed in South Africa and Saudi Arabia used convenience sampling techniques. In addition, Nepal and South Africa studies included women who sought abortion services and gave birth via elective cesarean section, respectively

The study revealed that primiparous mothers have a higher likelihood of being accompanied by their birth companion than their counterparts. This finding is supported by studies conducted in Brazil, Ghana, and Ethiopia [21,24,26]. The possible explanation is that primiparous mothers have negative expectations for childbirth, concern about powerlessness and loss of self-control during labor, which requires psychological support from companions [43,44].

The study revealed that complications during pregnancy significantly influence the utilization of a childbirth companionship. This finding is concurrent with studies done in Tanzania and Ethiopia [23,26]. This is because high-risk pregnancies need more support from both healthcare providers and companions to assist in decision-making and improve the outcome. However, this finding contradicts the study conducted in Kenya [24], which reported a higher likelihood of supportive care from family members of women without complications during delivery. The difference may be due to obstetric care providers’ need to keep mothers’ health information confidential and their fear of inappropriate companions’ behavior that might discourage the mother or interrupt the care provided for the mother.

Mothers’ knowledge of childbirth companionship significantly influences the utilization of a childbirth companionship. Besides, the mothers’ desire to have future childbirth companionship significantly impacts the utilization of birth companionship. This finding is consistent with the studies performed in Arba Minch and Debre Markos, Ethiopia [26,27]. This is because women who have a future desire to use a childbirth companion might have a good knowledge of the importance and role of having a companion during childbirth.

### Limitations of the study

Even though our study has the strength of using a mixed method to determine childbirth companionship utilization, it had a few limitations. First, the data were collected by self-reports, which can be exposed to social desirability and interviewer bias, which may affect the results to some extent. Second, because of the cross-sectional study design, it is difficult to ascertain the cause-and-effect relationship of variables. In addition, the qualitative data analysis may not reflect the perceptions of all women in the study.

## Conclusion

In this study, the magnitude of childbirth companionship utilization was low and below the WHO recommendation. Therefore, the study highlights the significance of acting on the health system, client, and provider-side factors, which are important to improve the implementation of respectful maternity care components at the facility level. In addition, the integration of respectful maternity care counseling into routine prenatal care, regardless of mothers’ pregnancy risk status, could enhance utilization. The findings also suggest that community-based awareness campaigns about the benefits and role of companions during childbirth influences mothers’ intention and enhance utilization rates. Moreover, addressing barriers to companionship utilization through healthcare provider training, developing clear facility-based guidelines, and improving health facilities’ infrastructure is crucial to enhance childbirth companionship and improve maternal health outcomes.

## Supporting information

S1 Data Dataset. (SAV)

## Abbreviations

ANC: Antenatal Care
AOR: Adjusted Odds Ratio
CI: Confidence Interval
COR: Crude Odds Ratio
EDHS: Ethiopian Demographic Health Survey
IDI: In-depth Interview
SPSS: Statistical Package Social Science
SD: Standard Deviation
SSA: Sub-Saharan Africa
WHO: World Health Organization.
